# Near- and Far-Field Optical Response of Eccentric Nanoshells

**DOI:** 10.1186/s11671-016-1796-8

**Published:** 2017-01-05

**Authors:** Ovidio Peña-Rodríguez, Pablo Díaz-Núñez, Vladimir Rodríguez-Iglesias, Luis Montaño-Priede, Antonio Rivera, Umapada Pal

**Affiliations:** 1 Instituto de Fusión Nuclear, Universidad Politécnica de Madrid, C/ José Gutiérrez Abascal 2, E-28006 Madrid, Spain; 2 Universidad Autónoma del Carmen, C/ 56 No. 4 Esq. Avenida Concordia, 24180 Ciudad del Carmen, Campeche Mexico; 3 Instituto de Física, Universidad Autónoma de Puebla, Apartado Postal J-48, 72570 Puebla, Puebla Mexico

**Keywords:** Eccentric nanoshells, FDTD method, Plasmon hybridization

## Abstract

We study the optical response of eccentric nanoshells (i.e., spherical nanoparticles with an eccentric spherical inclusion) in the near and the far field through finite-difference time-domain simulations. Plasmon hybridization theory is used to explain the obtained results. The eccentricity generates a far-field optical spectrum with various plasmon peaks. The number, position, and width of the peaks depend on the core offset. Near-field enhancements in the surroundings of these structures are significantly larger than those obtained for equivalent concentric nanoshells and, more importantly, they are almost independent of the illumination conditions. This opens up the door for using eccentric nanoshells in applications requiring intense near-field enhancements.

## Background

Metal nanoparticles (NPs) have unique electronic and optical properties that have spawned considerable interest. More precisely, they support collective oscillations of the free electrons, a phenomenon known as localized surface plasmon resonance (LSPR), with applications in several fields [1–5]. Some of those applications, like medical diagnostics [6], immunoassays [7, 8], and studies of living cells and bacteria [9, 10], require a fine control over the spectral properties (i.e., position and width) of the LSPR, which are susceptible to parameters like the size, shape, structure, and composition of the particles [11], along with the nature of dispersing dielectric medium. Metallic nanoshells [12, 13] and their variants [14–16] are well suited to provide this control because, even though they have a simple shape, they present a notable structural tunability of the plasmon frequencies [17, 18], as has been demonstrated both theoretically [12] and experimentally [19].

On the other hand, there are some applications such as surface-enhanced spectroscopies [9, 20, 21], thermal therapy of tumors [22], and thermoplasmonics [4, 23] that require an intense near field. Nanoshells are not equally attractive in this case because the reduction of thickness of the metal layer, which is required to tune the LSPR position, results in very weak near fields. Hence, different structures, like rough surfaces [24] and nanoparticle junctions [25], are usually preferred for these applications. The advantage of these structures comes from their ability to concentrate the light into small volumes, greatly enhancing the local electromagnetic (EM) field in this region, producing the so-called “hot spots” [26]. However, these structures have an important disadvantage: the position of the hot spots might be located in difficult to reach positions (e.g., in the interparticle gap or inside the irregularities). Spherical nanoshells with an eccentric core [27] or with surface defects [28] generate enhancements of the near electric field much more intense than that of the concentric ones. In other words, they combine the best of both worlds: a tunable LSPR coupled with large near-field enhancement. However, rough nanoshells have been far more studied in the literature [28–32] than the eccentric ones [27, 33, 34], probably because the latter are harder to synthesize [33].

In this paper, we have used finite-difference time-domain (FDTD) calculations [35, 36] to study the influence of geometrical parameters on the LSPR of eccentric nanoshells (ENs). We performed a simple expansion of Au dielectric function reported by Johnson and Christy [37] with a Drude term and five Lorentzian peaks, as has been described elsewhere [38]. It was found that the eccentricity shifts the plasmon energies and makes higher-order multipolar modes visible in the optical spectra. Moreover, near-field enhancements in the vicinity of ENs are much higher than those obtained around a concentric nanoshell. The obtained results are explained in terms of the theory of plasmon hybridization and they show that ENs have a great potential for applications requiring intense field enhancements like surface-enhanced spectroscopies, thermal therapy of tumors, and thermoplasmonics.

## Methods

### Calculation of the Optical Response

**Fig. 1:**
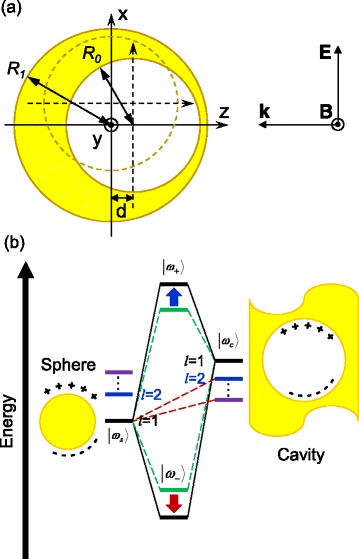
Schematic representation of the eccentric nanoshell structures. **a** Schematic representation of the simulated eccentric nanoshells with the core offset a distance *d* from the center of the shell. *R*_0_ and *R*_1_ represent the core and shell radii, respectively. **b** Energy level diagram representing the plasmon hybridization in the eccentric metallic nanoshells, resulting from the interaction of the sphere and the cavity plasmons. The *green dashed lines* illustrate the hybridization of sphere and cavity plasmons in a concentric nanoshell. For an eccentric nanoshell, there exists an interaction between primitive plasmon modes of all multipolar order (*red dashed lines*). This results in an extra shift for the eccentric nanoshell of the dipolar bonding and antibonding plasmons to the *red* and *blue*, respectively (represented by the *arrows* of the corresponding color)

Several theoretical approaches have been utilized to describe the light absorption and scattering by metallic nanostructures. For instance, the analysis of LSPR properties of metallic nanoparticles with spherical symmetry is usually done by means of the Mie theory [39, 40], whereas methods like FDTD [35, 36] are employed for other cases, e.g., for structures without spherical symmetry. The theory of plasmon hybridization [16] has also been developed to study the LSPR of metallic nanoparticles in terms of the interaction between the plasmons of metallic nanostructures with simpler forms (e.g., the LSPR of nanoshells is studied as the interaction of the modes in a sphere and a cavity). In this work, we have studied eccentric nanoshells with geometries as shown in Fig. 1a, using the computer software MEEP [41], which is an implementation of the FDTD method [35, 42]. A detailed description of the FDTD method can be reviewed elsewhere [35].

The dimensions of the ENs are set to 15 and 20 nm for the internal (*R*_0_) and external (*R*_1_) radii, respectively. The position of the core, which was set initially at the center of the sphere, is displaced by a distance, *d* (1 to 5 nm in steps of 1 nm) in the X, Y, and Z directions, respectively, to evaluate how the eccentricity affects the optical response in the far and near fields. The computational cell has a size of 160 × 160 × 160 nm^3^ with a spatial resolution of 0.66 nm and is surrounded by perfectly matched layers with a thickness of 200 nm to absorb the scattered waves. The structure is placed at the center of the cell. A broadband Gaussian source (200–900 nm) with the electric field polarized in the X direction is placed at the top of the cell and the EM wave propagates along the Z direction, interacting with the EN. The fields are allowed to evolve, and the simulation is terminated once |E|^2^ have decayed up to 10^−8^ at the bottom of the cell.

The far-field response is studied by means of the extinction efficiency factor, *Q*_ext_, which can be calculated from the extinction cross section, *C*_ext_, and the cross-sectional area projected onto a plane perpendicular to the incident beam, *A*, using the expression [40]:

1 $$ {Q}_{\mathrm{ext}}=\frac{C_{\mathrm{ext}}}{A}=\frac{C_{\mathrm{ext}}}{\pi\;{R}_1^2} $$

The extinction cross section represents the rate at which the incident radiation, *I*_inc_, is attenuated by the structure and, in a non-absorbing medium, it can be calculated as the sum of the absorption and scattering cross sections, *C*_abs_ and *C*_sca_ [40]:

2 $$ {C}_{\mathrm{ext}}={C}_{\mathrm{abs}}+{C}_{\mathrm{sca}}=\frac{W_{\mathrm{abs}}+{W}_{\mathrm{sca}}}{I_{\mathrm{inc}}} $$

where *W*_abs_ and *W*_sca_ represent the power absorbed and scattered by the structure, respectively.

The values of *I*_inc_ *, W*_abs_, and *W*_sca_ are calculated as the integral of the Poynting vector of the Fourier-transformed electric and magnetic fields at each frequency, *ω*, over a closed area to obtain a full spectrum in a single simulation [41]:

3 $$ W\left(\omega \right)=\Re {\displaystyle \oiint {\mathbf{E}}_{\omega}\times {\mathbf{H}}_{\omega}\;d\mathbf{S}} $$

The near-field response is evaluated by means of the field enhancement, |E|/|E_0_|, at the plasmon frequency of the structure. Each component of the complex electric field (*E*_*x*_, *E*_*y*_, and *E*_*z*_) is stored at each time step and every point of the space in the simulation cell. Once the fields, which are of the form of E = E (*x*, *y*, *z*, *t),* have been accumulated over the full simulation time, they are Fourier-transformed to obtain a full spectrum of the electric fields in the frequency domain, E = E (*x*, *y*, *z*, *ω*), for all the space considered in the simulation cell. This calculation is performed twice: firstly with the structure to obtain the fields scattered by the EN, |E| and then without the EN to obtain the incident field, |E_0_|, and normalize the previous result.

### Plasmon Hybridization Theory

Wu and Nordlander developed the plasmon hybridization theory to explain the LSPR behavior in ENs [27]. The energy level diagram for plasmon hybridization in the studied ENs is depicted in Fig. 1b. The LSPR of concentric metallic nanoshells can be viewed as the interaction between the plasmons of a sphere and a cavity. The hybridization of the plasmon of the sphere and the cavity creates two new plasmon oscillation modes, i.e., the higher energy (antibonding) mode |*ω*_+_〉 and the lower energy (bonding) mode |*ω*_−_〉, corresponding to the antisymmetric and symmetric interactions between the |*ω*_*s*_〉 and |*ω*_*c*_〉 modes, respectively.

Plasmon hybridization in an eccentric nanoshell is similar to that of its concentric counterpart, but the shift of the core introduces a very important difference: symmetry breaking eliminates the orthogonality between different-order modes. Hence, cavity and sphere modes of different multipolar indices can hybridize, forming bonding and antibonding modes that cannot exist in concentric nanoshells [27]. This additional interaction results in stronger hybridization and larger plasmon energy shifts of the dipolar bonding and antibonding plasmons to the red and blue, respectively. However, the more important effect of symmetry breaking lies in the fact that all the plasmon modes of the EN have a contribution from the optically active dipolar sphere plasmons. Consequently, several of the hybridized modes of the EN can be excited by the incoming light also in the dipole limit (i.e., for small particles, like the ones studied in this work, where higher-order modes are not expected to be visible) [27].

## Results and Discussion

**Fig. 2:**
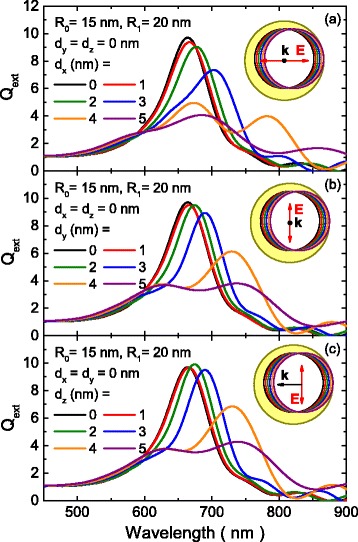
Extinction efficiency spectra as a function of the wavelength and eccentricity. Extinction efficiency spectra as a function of the wavelength for different values of eccentricity determined by the offset of the nanoshell core along the **a** X, **b** Y, and **c** Z axes

The extinction efficiency factor, *Q*_ext_, for all the studied cases is represented in Fig. 2 as a function of the core shift along the X (Fig. 2a), Y (Fig. 2b), and Z (Fig. 2c) axes. Obviously, the optical response of the concentric nanoshell (*d*_*x*_ = *d*_*y*_ = *d*_*z*_ = 0) is independent of the illumination conditions. As the eccentricity increases, the LSPR redshifts and widens, eventually splitting into two or even three peaks (for the larger offsets). This behavior is common to all the studied cases but is more prominent when the polarization of light is parallel to the direction of the core shift (Fig. 2a). Appearance of these additional peaks can be understood if we recall that plasmon modes with different multipolar orders cannot interact in concentric nanoshells due to the orthogonality condition. However, primitive plasmons with different indexes can interact when the symmetry is broken, producing hybridized plasmon modes that contain contributions from all multipolar orders. Moreover, the dipolar contribution in those hybrid modes means that they are optically active even in the dipole limit, which explains the new peaks that appear in the extinction spectra of the small ENs studied in this work.

Unlike the high-energy (>4.0 eV) modes reported by Wu and Nordlander [27], all the plasmon modes that appear in our results correspond to bonding modes. This difference comes from the fact that our dielectric function includes the interband transitions of gold, which damp the antibonding plasmon modes that lie in the same spectral region. The same effect is responsible for the widening of the bonding plasmon modes in our results in comparison to the modes reported by Wu and Nordlander [27]. On the other hand, it is remarkable that the spectra obtained for the polarizations perpendicular to the core shift direction are very similar, regardless the polarization/direction of the incident light, either perpendicular (Fig. 2b) or parallel to the offset (Fig. 2c). Finally, it should be noted that the extinction efficiency is somewhat attenuated as the core is shifted. This effect, common to all nanoshells [43], not only for eccentric ones, is related with the reduction of the metal layer. However, in spite of the reduction, the extinction efficiency is still intense enough for most applications. Moreover, the LSPR damping for a given plasmon shift would be lower in an eccentric nanoshell than in a concentric one because the additional shift introduced by the core makes it possible to obtain a given LSPR displacement with a lower *R*_0_/*R*_1_ ratio.

**Fig. 3:**
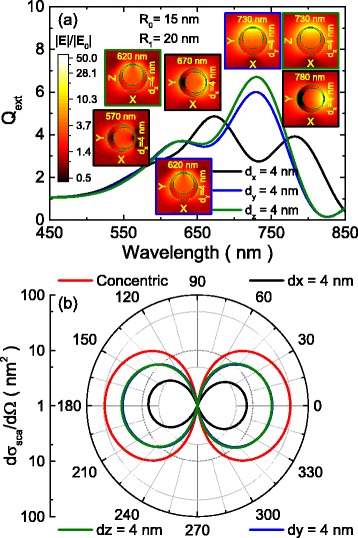
Extinction efficiency spectra and local electric field enhancements obtained for all the LSPR maxima. **a** Extinction efficiency spectra corresponding to the offset of 4 nm along all the Cartesian axes (polarization is fixed along the X axis). The *insets* are color maps representing the local electric field enhancements obtained for all the LSPR maxima. **b** Differential scattering cross section in the XZ plane at the LSPR maximum for the same structures and for the concentric nanoshell

A more detailed comparison between all the illumination conditions can be seen in Fig. 3. The optical extinction spectra obtained for the offset of 4 nm along all the Cartesian axes, together with insets representing the color maps of the local electric field enhancements obtained for all the LSPR maxima are depicted in Fig. 3a. All the trend discussed previously are clearly seen here: the spectra for the polarization perpendicular to the offset are very similar and exhibit two peaks, whereas the spectrum of the parallel polarization is redshifted and a third peak appears. In addition, the near-field enhancements are higher for the peaks located at larger wavelengths, even if the extinction efficiency is lower, as can be seen in the insets. Field enhancements (|E|/|E_0_|) are around 30 for those LSPR peaks, a twofold increase over a concentric nanoshell (not shown). The differential scattering cross section in the XZ plane at the LSPR maximum is shown in Fig. 3b for the same structures, along with the same quantity for the concentric nanoshell. It can be seen that the general behavior of scattering is not considerably affected by the eccentricity, and it is dominated by two large lobes in the forward and backward scattering directions. The important change that can be seen in Fig. 3b is the reduction of the total scattering, more marked when the core shift is parallel to the direction of the light polarization.

**Fig. 4:**
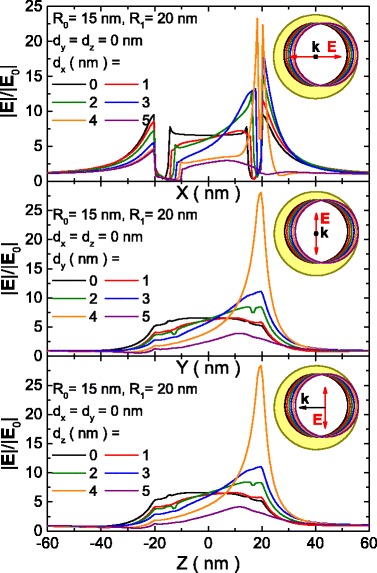
Near-field enhancement profiles along the eccentricity axis for different offsets. Near-field enhancement profiles along the eccentricity axis, calculated for nanoshells with the core displaced in the **a** X, **b** Y, and **c** Z axes

**Fig. 5:**
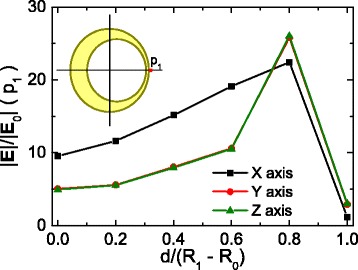
Near-field enhancement value in a point as a function of the eccentricity. Near-field enhancement value calculated in the point *p*_1_, as a function of the eccentricity in the three axes

The effect of the core shift in the near field is depicted in Figs. 4 and 5. Figure 4 shows the field enhancement profiles along each axis of the nanoshell, for the same direction of core shift. The distribution of field enhancement for zero offset is explained in Fig. 4. Along the polarization direction (Fig. 4a, X direction), it reaches its maximum in the vicinity of the shell, then goes to one inside it and is increased again inside the core, where it is almost constant. On the contrary, in a direction perpendicular to the polarization (Fig. 4b, c, Y and Z directions), the field enhancement increases near the shell until it reaches a certain value that is kept approximately constant inside the shell and core, without a clear maximum. Field enhancement is symmetrical with respect to the origin for the two latter cases.

The evolution of the field enhancement as a function of core shift is similar in all the cases, regardless of whether it is performed in the same direction or perpendicular to the polarization direction of the incident light. As the core shift increases, the value of |E|/|E_0_| becomes asymmetrical with the larger increases in the thinner side, whereas it decreases in the opposite side, reaching its maximum value for a core shift of 4 nm. For a more detailed analysis of the hot spot, Fig. 5 represents the value of |E|/|E_0_| in the point *p*_1_, located on the offset axis at 0.5 nm of the nanoshell surface as a function of core shift and illumination conditions. Field enhancement at this point reaches a value of about 20 when the core is shifted in the polarization direction of incident light and 25 for the axis perpendicular to the polarization direction. This means that the field is enhanced two- and sixfold, respectively, compared to the field enhancement produced around a concentric nanoshell. More importantly, similar values of the field enhancement are obtained for all the illumination conditions. This effect can have important implications for real-world applications, where it is nearly impossible to guarantee the alignment of the nanoshells with the polarization of light. The same is not true for the hot spots obtained in nanoparticle junctions that only reach the maximum value when the polarization of light is parallel to the axis of the structure [44, 45]. Hence, this effect alone makes eccentric nanoshells a very interesting candidate for applications requiring intense near-field enhancements without needing the specific orientation that require other plasmonic nanostructures.

## Conclusions

In this work, we have studied the evolution of the near- and far-field optical response of eccentric metallic nanoshells as a function of core offset. Plasmon modes in eccentric nanoshells are formed by hybridization of cavity and sphere plasmons of all multipolar orders, which is forbidden in concentric nanoshells by orthogonality conditions [27]. Hybridization becomes stronger on increasing the core offset, producing larger redshifts of bonding plasmons. Hybridized plasmon modes are composed of a mixture of all the primitive dipolar plasmons, which makes them optically active and gives rise to new LSPR peaks in the extinction spectrum. The electric field enhancements for resonant excitation of EN plasmons can be substantially higher than those obtained for their concentric counterparts. Moreover, the field enhancement obtained for the larger offsets is nearly independent of the illumination conditions. These two effects together make eccentric nanoshells very attractive for applications requiring intense near-field enhancements.

## Abbreviations

EM: Electromagnetic
ENs: Eccentric nanoshells
FDTD: Finite-difference time-domain
LSPR: Localized surface plasmon resonance
NPs: Nanoparticles

## References

[1] Ozbay E (2006). Plasmonics: merging photonics and electronics at nanoscale dimensions. Science.

[2] Lakowicz JR (2006). Plasmonics in biology and plasmon-controlled fluorescence. Plasmonics.

[3] Cialla D, März A, Böhme R (2011). Surface-enhanced Raman spectroscopy (SERS): progress and trends. Anal Bioanal Chem.

[4] Herzog JB, Knight MW, Natelson D (2014). Thermoplasmonics: quantifying plasmonic heating in single nanowires. Nano Lett.

[5] Wu J, Yu P, Susha AS (2015). Broadband efficiency enhancement in quantum dot solar cells coupled with multispiked plasmonic nanostars. Nano Energy.

[6] Allain LR, Vo-Dinh T (2002). Surface-enhanced Raman scattering detection of the breast cancer susceptibility gene BRCA1 using a silver-coated microarray platform. Anal Chim Acta.

[7] Hirsch LR, Jackson JB, Lee A (2003). A whole blood immunoassay using gold nanoshells. Anal Chem.

[8] Cui Y, Ren B, Yao J-L (2006). Synthesis of Ag_core_-Au_shell_ bimetallic nanoparticles for immunoassay based on surface-enhanced Raman spectroscopy. J Phys Chem B.

[9] Premasiri WR, Moir DT, Klempner MS (2005). Characterization of the surface enhanced Raman scattering (SERS) of bacteria. J Phys Chem B.

[10] Sikdar D, Rukhlenko ID, Cheng W, Premaratne M (2013). Optimized gold nanoshell ensembles for biomedical applications. Nanoscale Res Lett.

[11] Kelly KL, Coronado E, Zhao LL, Schatz GC (2003). The optical properties of metal nanoparticles: the influence of size, shape, and dielectric environment. J Phys Chem B.

[12] Peña O, Pal U, Rodríguez-Fernández L, Crespo-Sosa A (2008). Linear optical response of metallic nanoshells in different dielectric media. J Opt Soc Am B.

[13] Oldenburg SJ, Jackson JB, Westcott SL, Halas NJ (1999). Infrared extinction properties of gold nanoshells. Appl Phys Lett.

[14] Radloff C, Halas NJ (2004). Plasmonic properties of concentric nanoshells. Nano Lett.

[15] Wu D, Liu X (2009). Tunable near-infrared optical properties of three-layered gold-silica-gold nanoparticles. Appl Phys B.

[16] Prodan E, Radloff C, Halas NJ, Nordlander P (2003). A hybridization model for the plasmon response of complex nanostructures. Science.

[17] Oldenburg SJ, Averitt RD, Westcott SL, Halas NJ (1998). Nanoengineering of optical resonances. Chem Phys Lett.

[18] Qian J, Sun Y-D, Li Y-D (2015). Nanosphere-in-a-nanoegg: damping the high-order modes induced by symmetry breaking. Nanoscale Res Lett.

[19] Schwartzberg AM, Olson TY, Talley CE, Zhang JZ (2006). Synthesis, characterization, and tunable optical properties of hollow Gold nanospheres. J Phys Chem B.

[20] Otto A, Mrozek I, Grabhorn H, Akemann W (1992). Surface-enhanced Raman scattering. J Phys Condens Matter.

[21] Park H-J, Vak D, Noh Y-Y (2007). Surface plasmon enhanced photoluminescence of conjugated polymers. Appl Phys Lett.

[22] Hirsch LR, Stafford RJ, Bankson JA (2003). Nanoshell-mediated near-infrared thermal therapy of tumors under magnetic resonance guidance. Proc Natl Acad Sci U S A.

[23] Harris N, Ford MJ, Cortie MB (2006). Optimization of plasmonic heating by gold nanospheres and nanoshells. J Phys Chem B.

[24] Fu EG, Caro M, Zepeda-Ruiz LA (2012). Surface effects on the radiation response of nanoporous Au foams. Appl Phys Lett.

[25] González-Rubio G, González-Izquierdo J, Bañares L (2015). Femtosecond laser-controlled tip-to-tip assembly and welding of gold nanorods. Nano Lett.

[26] Wei H, Xu H (2013). Hot spots in different metal nanostructures for plasmon-enhanced Raman spectroscopy. Nanoscale.

[27] Wu Y, Nordlander P (2006). Plasmon hybridization in nanoshells with a nonconcentric core. J Chem Phys.

[28] Peña-Rodríguez O, Pal U (2011). Enhanced plasmonic behavior of incomplete nanoshells: effect of local field irregularities on the far-field optical response. J Phys Chem C.

[29] Shi W, Sahoo Y, Swihart MT, Prasad PN (2005). Gold nanoshells on polystyrene cores for control of surface plasmon resonance. Langmuir.

[30] Wang H, Goodrich GP, Tam F (2005). Controlled texturing modifies the surface topography and plasmonic properties of Au nanoshells. J Phys Chem B.

[31] Sauerbeck C, Haderlein M, Schürer B (2014). Shedding light on the growth of gold nanoshells. ACS Nano.

[32] Xie H, Larmour IA, Smith WE (2012). Surface-enhanced Raman scattering investigation of hollow gold nanospheres. J Phys Chem C.

[33] Wang H, Wu Y, Lassiter B (2006). Symmetry breaking in individual plasmonic nanoparticles. PNAS.

[34] Zhang J, Zayats A (2013). Multiple Fano resonances in single-layer nonconcentric core-shell nanostructures. Opt Express.

[35] Taflove A, Hagness SC (2005). Computational electrodynamics: the finite-difference time-domain method.

[36] Yee K (1966). Numerical solution of initial boundary value problems involving Maxwell’s equations in isotropic media. IEEE Trans Antennas Propag.

[37] Johnson PB, Christy RW (1972). Optical constants of the noble metals. Phys Rev B.

[38] Hao F, Nordlander P (2007). Efficient dielectric function for FDTD simulation of the optical properties of silver and gold nanoparticles. Chem Phys Lett.

[39] Mie G (1908). Beiträge zur optik trüber medien, speziell kolloidaler metallösungen. Ann Phys.

[40] Bohren CF, Huffman DR (1998) Absorption and scattering of light by small particles. Weinheim: Wiley-Interscience.

[41] Oskooi AF, Roundy D, Ibanescu M (2010). Meep: a flexible free-software package for electromagnetic simulations by the FDTD method. Comput Phys Commun.

[42] Taflove A, Oskooi A, Johnson SG (2013) Advances in FDTD computational electrodynamics: photonics and nanotechnology. Boston: Artech House.

[43] Peña-Rodríguez O, Pal U, Kumar CSSR (2013). Exploiting the tunable optical response of metallic nanoshells. UV-VIS and Photoluminescence Spectroscopy for Nanomaterials Characterization.

[44] Nordlander P, Oubre C, Prodan E (2004). Plasmon hybridization in nanoparticle dimers. Nano Lett.

[45] Hao E, Schatz GC (2004). Electromagnetic fields around silver nanoparticles and dimers. J Chem Phys.

